# Incidence of Attention Deficit Hyperactivity Disorder (ADHD) Diagnoses in Navarre (Spain) from 2003 to 2019

**DOI:** 10.3390/ijerph18179208

**Published:** 2021-08-31

**Authors:** Leire Leache, Olast Arrizibita, Marta Gutiérrez-Valencia, Luis Carlos Saiz, Juan Erviti, Julián Librero

**Affiliations:** 1 Unit of Innovation and Organization, Navarre Health Service, 31003 Pamplona, Navarre, Spain; lleachea@navarra.es (L.L.); mgutierv@navarra.es (M.G.-V.); lsaizfer@navarra.es (L.C.S.); 2 NNBi, 31191 Pamplona, Navarre, Spain; olastt1@gmail.com; 3 Navarre Institute for Health Research (IdiSNA), 31008 Pamplona, Navarre, Spain; 4 Therapeutics Initiative, Department of Anesthesiology, Pharmaceuticals and Therapeutics, University of British Columbia, Vancouver, BC V6T 1Z4, Canada; juan.ervitilopez@ubc.ca; 5 Navarrabiomed Biomedical Research Centre, Navarre Institute for Health Research (IdiSNA), Complejo Hospitalario de Navarra, Public University of Navarre (UPNA), 31008 Pamplona, Navarre, Spain

**Keywords:** attention deficit hyperactivity disorder, hyperactivity, inattention, behavior disorder

## Abstract

(1) Background: Attention deficit hyperactivity disorder (ADHD) constitutes one of the leading mental health and behavioral disorders in childhood and adolescence. The main objective of this study was to analyze the time trend in the incidence of ADHD diagnoses in Navarre (Spain) from 2003 to 2019 in children and adolescents from 5 to 19 years old. Additionally, the seasonal trends of ADHD incidence and ADHD prevalence were determined. (2) Methods: A population-based observational retrospective study, which included people born between 1991 and 2011 and who attended compulsory education between 2007 and 2017 in Navarre (Spain), was carried out with data from both the Education and Health Department databases. (3) Results: The incidence rate increased from 4.18 cases per 1000 person-years in 2003 to 7.43 cases per 1000 person-years in 2009, before decreasing progressively to 2.1 cases per 1000 person-years in 2019. A peak incidence rate at 7–8 years of age was observed, which is consistent across the study period and for both genders. Males were more than twice as likely to be diagnosed with ADHD than females, with similar time trends in both. A seasonal pattern in ADHD diagnosis was found, with peaks in February–March and the lowest rates in the summer months. Inattentive cases were much more frequent than hyperactive cases, whereas combined cases remained low across the study period. (4) Conclusions: In this age-period-cohort analysis, a clear period and age effect was observed. We found a decreasing trend in the ADHD incidence rate since 2015. Further research is needed to confirm whether a change of trend is occurring globally.

## 1. Introduction

Attention deficit hyperactivity disorder (ADHD) constitutes one of the leading mental health and behavioral disorders in childhood and adolescence. The American Psychiatric Association’s Fifth Edition of the Diagnostic and Statistical Manual of Mental Disorders (DSM-5) defines ADHD in children younger than age 17 years as the presence of six or more symptoms in either the inattentive or hyperactive and impulsive domains, or both [1]. The etiology of ADHD is uncertain. The diagnosis is mainly based on clinical signs and symptoms, as there is no sound evidence for specific analytical alterations. Therefore, even with clear-cut diagnostic criteria, there is a potential risk of overdiagnosis and underdiagnosis [2].

ADHD shows a marked heterogeneity at clinical, aetiological, and pathophysiological levels [2]. The likelihood of being diagnosed with ADHD has been associated with multiple factors, such as the diagnostic classification used [3,4], being born in the later months of the school year as compared to the early months [5,6], socio-economic status [7,8,9], race/ethnicity [10], the characteristics of the home environment [7], and parental history of ADHD diagnosis [8].

Gender differences in ADHD diagnosis have also been documented, with girls being less likely to be diagnosed [11]. In childhood, the prevalence of ADHD in boys is 2–2.5 times higher than its prevalence in girls, and by adulthood this ratio becomes closer to equal [12].

An association between the month of birth and the likelihood of being diagnosed with ADHD has also been identified [5,6,13]. However, the potential impact of seasonality in ADHD diagnosis has not yet been investigated.

Several studies and systematic reviews have been conducted to estimate the prevalence of ADHD, reporting highly variable rates, which may be attributable to differences in the population studied, the diagnostic criteria used, sources of information, and the methodology, among other factors [14]. Systematic reviews analyzing the evidence published between the 1970s and the first decade of the 2000s obtained an ADHD prevalence of 5.3% and 7.2% in children and adolescents up to 18 years of age worldwide [15,16]. Another review that included studies published between 1985 and 2012 estimated a worldwide ADHD prevalence of 3.4% (95% CI 2.6–4.5) for children and adolescents in the same age range [17]. A recently published review covering the period 2003–2020 reported a prevalence of 3.7% (95% CI 2.3–5.7%) in children and adolescents up to 18 years of age from high-income countries [18]. The variability observed in the ADHD prevalence estimates may also be influenced by the studied time period; therefore, analyzing the trends in ADHD diagnoses over time is of particular interest.

Differences in the ADHD prevalence have also been identified in the different geographic regions [19]. The ADHD prevalence in people under 18 years of age in Spain would be 6.8% (95% CI 4.9–8.8%) according to a review published in 2012 [20]. However, scarce data are available regarding the tendency of ADHD diagnoses over time in Spain. 

Most of the studies analyzing the ADHD incidence in the long term reported an increasing trend in the incidence rate over the course of the years studied [21,22,23,24,25]. In contrast, a study including children and adolescents aged 4–17 years from Catalonia (Spain) did not observe a statistically significant increase in the incidence of ADHD diagnoses between 2009 and 2017 [26].

ADHD medication use also experienced an increasing tendency over the years, with rates varying in the different countries. A population-based study including 13 countries worldwide found that the prevalence of ADHD medication use in children aged 3–18 years ranged between 0.3% and 6.7% in 2010, with an increasing tendency over time both in children and adults. North America had the highest prevalence of ADHD medication [27]. A seasonal pattern on the use of ADHD medication has been observed in many published studies, with use being lower during the summer months [28,29].

Knowledge about the trends in ADHD diagnoses is essential to organize health resources with a forward-looking approach. To our knowledge, few studies have been carried out with the aim to analyze the time trend incidence of ADHD in Spain, and none of those studies included an extended period of time. Additionally, the potential impact of seasonality in ADHD diagnosis has not been evaluated to date. Therefore, the main objective of this study was to analyze the time trend in the incidence of ADHD diagnoses from 2003 to 2019 according to calendar year, patients’ age at the time of ADHD diagnosis, and gender. The secondary objectives were to analyze: (1) the incidence of ADHD diagnoses according to the ADHD presentation; (2) the monthly variation of the ADHD incidence (seasonal trend), also according to the clinical setting where the ADHD diagnosis was registered; and (3) the time trend of the prevalence of the ADHD diagnosis, also differentiating by the patients’ age and gender.

## 2. Materials and Methods

### 2.1. Data Source

An observational retrospective study was carried out with data from the database of the Education Department in Navarre (EDUCA) and the Navarre Health Service population database (BARDENA). Both databases were linked, matching and anonymizing the subjects. Inconsistencies were solved by creating decision algorithms and using automatic probabilistic methods.

### 2.2. Participants and Follow-Up

The study included people who were born between 1991 and 2011, were entitled to public health services, and who also attended at least one annual course of compulsory education between 2007 and 2017 in public schools or centers subsidized by the Government of Navarre (in this period there was only one non-subsidized private center in Navarre). Students who held health insurance different from that provided by the National Health System (primarily public mutualities) were excluded from the study.

The follow-up of children started at the age of 5 years. Follow-up ended in the event of death or the end of compulsory education or health data availability (whichever occurred later), and ended, in any case, at the age of 20 years or on 20 November 2019 (end of the study period).

### 2.3. ADHD Diagnostic Codes and Study Variables

ADHD diagnoses from both Primary Care [through the International Classification of Primary Care-2 (ICPC-2)] and Specialized Care [through the 10th Revision of the International Classification of Diseases (ICD-10)] were considered (Table S1). Two reviewers (LL and LCS) independently carried out an individual validation of the ADHD diagnoses by revising the literal text associated with the ICPC codes. Unconfirmed or doubtful cases of ADHD were excluded. The diagnosis date was the date when the ADHD diagnosis was first registered either in Primary or Specialized Care.

Registered variables included the patients’ age, gender, nationality, ADHD diagnosis, date of diagnosis, the clinical setting in which the ADHD was diagnosed (Primary or Specialized Care), and the ADHD presentation profile (hyperactive/impulsive, inattentive, combined or undetermined/unknown).

### 2.4. Data Analyses

Median and interquartile range (IQR) were calculated for continuous variables, and proportion and confidence interval for dichotomous variables.

#### 2.4.1. ADHD Incidence

The ADHD incidence rate was calculated as the ratio of incident cases per 1000 person-years of exposure. The ADHD incidence was described according to the patients’ age at the time of the ADHD diagnosis, their gender, and the calendar year in biennial groups. Results were also provided according to the ADHD profile. Age-period-cohort (APC) regression models were performed to investigate how ADHD incidence was affected by age at the time of ADHD diagnosis, period of ADHD diagnosis and cohort of birth [30].

Additive time series decomposition and Poisson regression models were developed to describe the evolution of ADHD incidence according to gender, age, calendar month and year-period of ADHD diagnosis.

Decomposition of the monthly time series of the standardized ADHD incidence rate was represented according to the calendar year, showing separately the observed cases, trend, seasonality and the random error. Results were also shown according to the clinical setting of the ADHD diagnosis. The seasonality of the incidence of ADHD in women and men was analyzed using box diagrams showing the incidence of new diagnoses throughout the different months of the year.

#### 2.4.2. ADHD Prevalence

The prevalence of ADHD diagnosis was provided for four different time points across the study period: 2007, 2011, 2015 and 2019. Results were also shown according to the patients’ age and gender. As for the analyses of the prevalence, the patients’ follow-up and, where possible, the closing/ending of the ADHD diagnoses were considered.

All the analyses were made using R software (fastLink, Epi and apc packages) [30,31,32].

### 2.5. Study Approval

The study protocol was approved by the Ethics Committee of the Government of Navarre, Spain, in 2016 (project 2016/73).

## 3. Results

During the study period, 124,582 individuals were part of the cohort (1,167,478 person-years), of which 51.2% were males. The majority were Spanish (82.8%), followed by Latin Americans (7.6%). The median follow-up was 11 years (IQR: 7–15). A total of 7391 ADHD cases were detected (5363 males and 2028 females).

**Figure 1:**
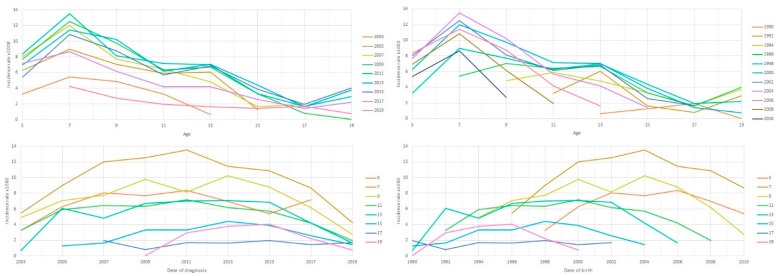
Rates of ADHD (per 1000 person-years) in Navarre 2003–2019 in ages 5–19 years. (**Top left**): age-specific rates by period of diagnosis. (**Top right**): age-specific rates by date of birth. (**Bottom left**): period-specific rates by age. (**Bottom right**): cohort-specific rates by age.

The ADHD cases, person-years and incident rate in biennial groups are shown in Table S2. The maximum incidence rate was reached in 2009. The rate increased from 4.18 cases per 1000 person-years in 2003 to 7.43 in 2009, and decreased progressively to 2.21 cases per 1000 person-years in 2019. The incidence of ADHD diagnosis according to gender, age at the time of ADHD diagnosis and calendar year is shown in Table 1. The incidence rate was significantly higher in males than in females for most of the age range and analyzed years, except in some upper extreme ages in which significant differences were not observed; this was probably due to the lower number of participants in these age ranges. Throughout the study period, the highest incidence rate occurred in children aged 7–8 years. The overall incidences of ADHD diagnosis at age-specific rates by period of diagnosis or by date of birth, and at period/cohort specific rates by age, are represented in Figure 1. Figure S1 represents the overall incidence of ADHD according to age at ADHD diagnosis and calendar year, with the findings differentiated by gender.

**Figure 2:**
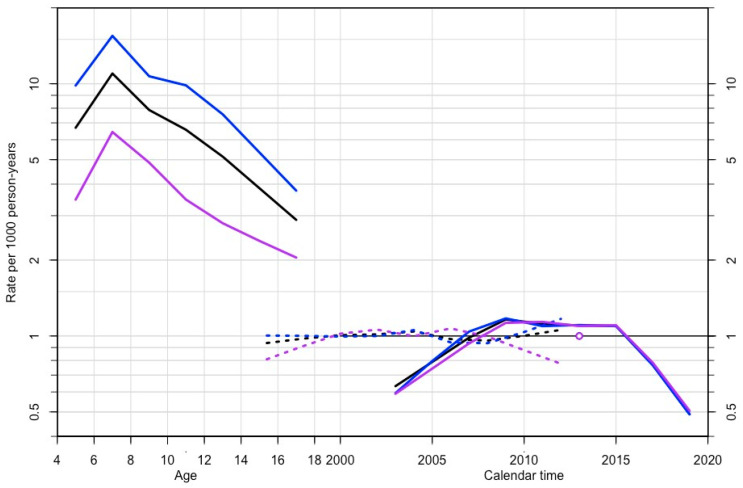
Age-period-cohort model. On the left, age-effect in rate scale; on the right, cohort (broken lines) and period (full lines) effect in risk ratio scale. Male (blue line), female (purple), both (black).

The age-period-cohort model is shown in Figure 2 and the results for the goodness of fit for the models are shown in Table S3. The Poisson regression analysis used to model the effect of gender and the various time components (age, month and year period) on the ADHD incidence is shown in Figure S2 and Table S4. A period effect is evidenced, with a lower risk of ADHD diagnosis at the extremes of the period distribution, and a higher risk between 2007 and 2015. However, a marked cohort effect is not observed. Age effects showed a medium rate at the age of 5, increasing to the highest risk at the age of 7, and a progressively decreasing rate from there to older ages. These effects were similar in males and females. An effect of gender (incidence rate ratio male:female 2.55:1), age (maximum at 7 years old), period (maximum in 2013), and season (maximum in February and March) on the risk of being diagnosed with ADHD was observed.

**Figure 3:**
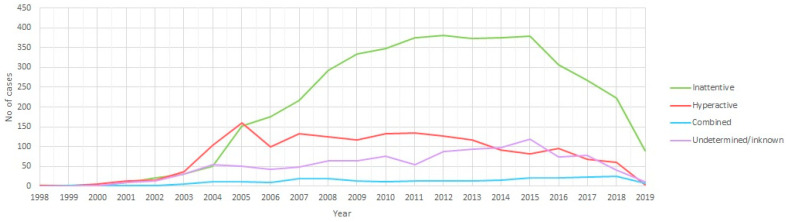
Incidence of Attention deficit hyperactivity disorder (ADHD) diagnosis by calendar year and ADHD profile (both males and females) (2019 data up to 20 November).

The incidence rate of ADHD diagnosis by calendar year and ADHD profile is shown in Figure 3. Inattentive cases were much more frequent than hyperactive or combined cases after 2005. From this time point on, hyperactive cases remained stable or decreased, while inattentive cases continued increasing until 2012, remained stable and then started decreasing in 2015.

**Figure 4:**
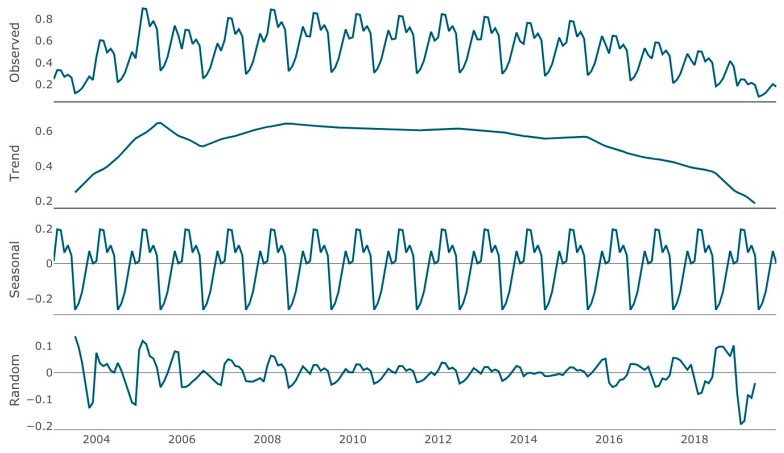
Decomposition of the monthly time series of the standardized Attention deficit hyperactivity disorder (ADHD) incidence rate by calendar year (both males and females).

Figure 4 represents the decomposition of the monthly time series of the standardized ADHD incidence rate by calendar year. The observed seasonal pattern remained very stable throughout the entire study period, except at the initial and final extremes. Removing the seasonal effect, the trend showed a progressive increase in incidence from 2003 to 2009 (with a decrease in 2007 and a subsequent increase), a plateau until 2015 and a decrease until the end of the study period.

The incidence of new diagnoses throughout the different months in males and females are shown in Figure S3. The seasonal pattern was similar in both genders, with peaks in February and March and the lowest values in July–September, coinciding with the summer academic break. 

Data according to the clinical setting of the ADHD diagnosis are shown in Figure S4. Specialized Care did not diagnose ADHD until 2011, with a significant increase in diagnoses in 2014. Until then, most diagnoses were made in Primary Care, and after the incursion of Specialized Care they continued with the same rate of diagnoses.

Data for the prevalence of ADHD diagnoses are shown in Table S5 and Figure S5. The overall prevalence was 2.4% in 2007, 4.1% in 2011, 5.3% in 2015 and 5.9% in 2019. The prevalence progressively increased from 2007 to 2019, although incidence has decreased in recent years. Consequently, the age with the highest prevalence has varied over time, being 11 years in 2007 and 2011, 15 years in 2015, and 19 years in 2019. The ADHD prevalence was significantly higher in males than in females except in some extreme age ranges.

## 4. Discussion

This observational study with a 16-year follow-up showed remarkable findings on the incidence of ADHD in children and adolescents in a Spanish region, regarding temporal changes and the effect of age, gender, or the setting of diagnosis. We observed a clear period, gender and age effect. 

ADHD incidence increased by 77% from 2003 to 2009. After a plateau period, it started decreasing in 2015, reaching rates in 2019 even lower than those at the beginning of the study. So far, most epidemiological studies on ADHD incidence have shown a continuously increasing temporal trend, which is not in line with the decreasing trend observed in Navarre over the last few years [21,22,23,24,25]. In contrast, and similarly to our study, a large population-based study in Israel found a dramatic increase in the rate of ADHD diagnoses from 2005, peaking in 2011, before declining in 2014 [33]. A population-based study in Catalonia (Spain) between 2009 and 2017 showed similar ADHD incidence rates, and did not find a significant increase during the study period [26]. These differences could be explained by the fact that these studies do not cover the latter period, or by differences in ADHD diagnostic criteria, the studied population or methodological procedures.

The shifts in temporal trends of ADHD incidence observed in our study may be in part related to several contextual factors. The approval of ADHD medication could indirectly have had an impact on the increase in the new ADHD diagnoses observed in the first period. Moreover, the increasing awareness among the parents, teachers and healthcare professionals about ADHD occurrence at early years may explain this tendency. However, the efforts made in recent years for the early detection of signs of inattention and hyperactivity in children could have facilitated the adoption of early measures both in the childrens’ home and school environments, avoiding the need for intervention by health professionals and thus contributing to a deceleration of identification of new confirmed ADHD cases.

The ADHD prevalence increased over the years from 0.15% to 8.74% according to age and calendar year. The age with the highest prevalence also increased as the study period progressed; this was due to the decrease in incidence rate in recent years.

A systematic review including studies published between 1980 and 2011 showed an ADHD pooled-prevalence of 6.8% in children and adolescents under 18 years from Spain [20], which is slightly higher than our results, although the review did not analyze data from the most recent years. A further study showed that the prevalence of ADHD in children aged 4 to 17 years from Catalonia (Spain) was 4.1% in 2017, which is lower than was observed in our study [26]. Differences in the source of the data, diagnostic criteria or sociodemographic factors could explain the differences in the prevalence estimates.

The age effect, with a peak incidence rate at 7–8 years and progressively decreasing afterwards, is consistent across the study period. Previous studies in different locations found a similar distribution, with ADHD diagnosis most commonly observed during primary school years (especially between 7 and 9 years) [23,24,34]. It is unknown if this disorder commonly develops or first manifests around this time or if a preexisting ADHD is more likely to be recognized as a problem during a child’s early years of formal schooling, a time when it has a more disruptive impact [23].

A clear gender effect was also found, with a generally higher ADHD incidence and prevalence in males. These findings are consistent with results from previous studies that have typically found ADHD to be 2–4 times more common in males than in females [21,23,26,34].

A well-defined seasonal pattern was observed in the diagnosis of ADHD, with peaks of incidence in February and March and lower values in July–September, coinciding with the summer school holidays. Although many published studies have shown a seasonal pattern on the ADHD medication use (use being lower during the summer months) [28,29], we have found the first evidence of a seasonal trend in ADHD diagnosis. This reinforces the idea that ADHD diagnosis is linked to the disruption it produces in the school environment or in academic performance. Related to this, previous studies have found that the youngest children in their grades were more likely to receive an ADHD diagnosis [5,6,13].

As for the gender factor, trends are very similar throughout the periods, months of the year and age at diagnosis, but always with higher rates in males. Some studies have found the same pattern [23,24], although others, like a population-based study in Denmark, found that ADHD incidence peaked earlier in boys than in girls (8 vs. 17 years of age) [34].

Since 2005, inattentive cases have been much more frequent than hyperactive cases, and their incidence rate continued increasing until 2012. Combined cases remained low across the study period. This relevant finding seems to confirm that current ADHD diagnoses differ to a high extent from the original hyperactive disorder as defined in the late 1960s by the DSM-II, showing a closer connection to academic performance at present.

Information bias in our sources may exist due to a possible absence of ADHD diagnosis records when those records have been made in private health centers. This motivated the exclusion of the people whose diagnosis, treatment and/or monitoring were carried out in private health centers. However, even in these cases, the patient’s general practitioner or Primary Care pediatrician of the National Health System usually includes this diagnosis in the clinical record. The diagnoses of Primary Care may have a closing or completion date, while those of Specialized Care always remain active in the system. This can lead to some inaccuracies in the prevalence data, although the impact is limited, given that not many diagnoses are expected to be reversed during adolescence. Another potential limitation is related to the fact that, according to our protocol design, some population ages are not represented in the extreme dates of the considered time interval. However, any potential bias was minimized by the use of incidence rates within each age.

Despite these limitations, to our knowledge, this is the first age-period-cohort analysis to assess temporal changes in the incidence of ADHD, and one of the first to study ADHD incidence over an extended time period in Spain, including the most recent years. Spain’s National Health System provides universal access to health services for all children and adolescents, so the cohort includes most of the population, regardless of their socioeconomic status, place of birth or racial/ethnic identification. The electronic health record of the National Health System is probably the most ideal of all data sources in Spain to study temporal changes of a medical condition such as ADHD. Moreover, the diagnoses are very reliable, as the codes were individually validated, and unconfirmed or doubtful cases of ADHD were excluded. We also stratified the analyses by ADHD subtypes (predominantly inattentive, predominantly hyperactive/impulsive, and combined patterns), which has not been reported in most ADHD incidence studies. Finally, a substantial number of participants, together with a long follow-up period, provided the obtained results with adequate accuracy.

## 5. Conclusions

In this age-period-cohort analysis of a population-based study, a clear period and age effect was observed. The period effect showed an incidence increase in the first years, followed by a plateau period, and then a progressive decrease in the last years. Further research should confirm whether there has been a global change in trend in recent years. The age effect, with a peak incidence rate at 7–8 years of age is consistent across the study period and for both genders. The well-known predominance of ADHD diagnoses in males was also observed, along with similar time trends in males and females. Clear evidence for a seasonal pattern in ADHD diagnosis was found for the first time, with peaks in February–March and the lowest rates in the summer months. Inattentive cases were much more frequent than hyperactive cases, and combined cases remained low across the study period.

## Figures and Tables

**Table 1:** Incidence rate of Attention deficit hyperactivity disorder (ADHD) diagnosis according to calendar year, patients’ age at the time of ADHD diagnosis, and gender.

Calendar Year	Age at the Time of ADHD Diagnosis (Years)	Incidence Rate (Per 1000 Persons)			*p*-Value
		Overall	Males	Females	
2003	5	3.26	4.76	1.69	<0.01
	7	5.42	8.53	2.26	<0.01
	9	4.85	8.26	1.22	<0.01
	11	3.27	5.25	1.06	<0.01
	13	0.63	1.18	0.00	--
	15	--	--	--	--
	17	--	--	--	--
	19	--	--	--	--
2005	5	6.29	9.90	2.52	<0.01
	7	8.99	13.08	4.77	<0.01
	9	7.05	11.07	3.02	<0.01
	11	5.91	9.30	2.35	<0.01
	13	6.07	10.64	1.07	<0.01
	15	1.27	2.41	0.00	--
	17	--	--	--	--
	19	--	--	--	--
2007	5	8.07	11.71	4.35	<0.01
	7	12.02	17.15	6.77	<0.01
	9	7.73	11.69	3.73	<0.01
	11	6.41	9.61	3.26	<0.01
	13	4.81	8.01	1.51	<0.01
	15	1.65	2.35	0.91	0.07
	17	1.96	2.50	1.36	1
	19	--	--	--	--
2009	5	7.69	11.37	3.82	<0.01
	7	12.50	17.47	7.54	<0.01
	9	9.77	13.15	6.35	<0.01
	11	6.34	9.96	2.74	<0.01
	13	6.67	9.54	3.89	<0.01
	15	3.29	3.76	2.82	0.33
	17	0.81	1.04	0.56	0.51
	19	--	--	--	--
2011	5	8.34	12.48	4.11	<0.01
	7	13.49	19.00	7.83	<0.01
	9	8.16	10.14	6.22	<0.01
	11	7.16	10.16	4.17	<0.01
	13	6.99	10.51	3.53	<0.01
	15	3.31	4.62	2.04	<0.01
	17	1.69	2.45	0.92	0.03
	19	2.95	3.61	2.24	0.36
2013	5	6.96	9.52	4.26	<0.01
	7	11.43	15.15	7.68	<0.01
	9	10.21	14.41	5.99	<0.01
	11	6.19	9.52	2.94	<0.01
	13	7.08	10.74	3.48	<0.01
	15	4.42	6.47	2.44	<0.01
	17	1.65	2.01	1.31	0.29
	19	3.75	6.00	1.50	<0.01
2015	5	5.38	8.22	2.42	<0.01
	7	10.84	15.41	6.09	<0.01
	9	8.78	11.90	5.67	<0.01
	11	5.69	8.47	2.92	<0.01
	13	6.85	10.49	3.37	<0.01
	15	3.87	4.61	3.16	0.1
	17	1.96	2.27	1.67	0.42
	19	4.01	5.96	2.19	<0.01
2017	5	7.16	11.06	3.04	<0.01
	7	8.66	13.05	4.15	<0.01
	9	6.16	7.94	4.33	<0.01
	11	4.19	5.82	2.58	<0.01
	13	4.18	6.17	2.22	<0.01
	15	2.59	3.12	2.10	0.18
	17	1.45	1.34	1.55	0.85
	19	2.19	3.19	1.26	0.07
2019	5	--	--	--	--
	7	4.23	5.91	2.49	0.03
	9	2.71	4.27	1.14	<0.01
	11	1.96	2.76	1.16	0.14
	13	1.64	2.21	1.08	0.2
	15	1.43	2.45	0.50	0.03
	17	1.71	1.87	1.57	1
	19	0.76	0.86	0.69	1

## Data Availability

Data were obtained from the Navarre Health Service and the Education Department in Navarre. Restrictions apply to the availability of these data.

## Supplementary Materials

The following are available online at https://www.mdpi.com/article/10.3390/ijerph18179208/s1. Table S1: Attention deficit hyperactivity disorder (ADHD) diagnostic codes. Table S2: Biennial Attention deficit hyperactivity disorder (ADHD) cases, person-years and incidence rate. Table S3: Goodness of fit of the age-period-cohort model. Table S4: Modeling of the effect of age, gender, year period and month on the incidence rate ratio of Attention deficit hyperactivity disorder (ADHD) (Poisson regression). Table S5: Prevalence of Attention deficit hyperactivity disorder (ADHD) diagnosis according to calendar year, patients’ age at the time of ADHD diagnosis and gender. Figure S1: Incidence of Attention deficit hyperactivity disorder (ADHD) diagnosis according to age at the time of ADHD diagnosis and calendar year. Top: MALES. Bottom: FEMALES. Figure S2: Modeling of the effect of age, gender, year period and month on the incidence of Attention deficit hyperactivity disorder (ADHD) (Poisson regression). Reference categories: year 2003, month 01 (January), age 5. Estimates >1 represent higher incidences. Figure S3: Seasonality of Attention deficit hyperactivity disorder (ADHD) incidence. Left: FEMALES. Right: MALES. Figure S4: Decomposition of the monthly time series of the standardized Attention deficit hyperactivity disorder (ADHD) incidence rate by calendar year (both males and females) (2019 data up to 20 November). Top: ADHD diagnoses from PRIMARY CARE. Bottom: ADHD diagnoses from SPECIALIZED CARE. Figure S5: Prevalence of Attention deficit hyperactivity disorder (ADHD) diagnosis according to calendar year and patients’ age at the time of ADHD diagnosis (both males and females).
