# Supplementary material for: Incidence of Attention Deficit Hyperactivity Disorder (ADHD) Diagnoses in Navarre (Spain) from 2003 to 2019
Source: Int J Environ Res Public Health. 2021 Aug 31;18(17):9208. doi: 10.3390/ijerph18179208 (PMC8431029; doi:10.3390/ijerph18179208)
Supplement: Supplementary file 1 [file ijerph-18-09208-s001.zip › ijerph-1292075-supplementary.pdf]

Table S1. Attention deficit hyperactivity disorder (ADHD) diagnostic codes

Classification system	ADHD diagnostic code
International Classification of Primary Care (ICPC)	P81: Hyperactivity disorder[a]
	P22: Behavioral signs/symptoms in children. Behavioral or conduct problems[b]
	P23: Behavioral signs/symptoms in adolescents[b]
	P24: Specific learning problems[b]
10th Revision of the International Classification of Diseases (ICD-10)	F90.0: Attention deficit hyperactivity disorder, with predominance of inattentive type
	F90.1: Attention deficit hyperactivity disorder, with predominance of hyperactive type
	F90.8: Attention deficit hyperactivity disorder, other type
	F90.9: F90.8: Attention deficit hyperactivity disorder, type not specified

[a]Cases in which the literal text associated to the diagnostic code corresponded to ADHD diagnoses were only included.

[b]Cases in which the literal text associated to the diagnostic code included the following terms in Spanish were only considered: "TDA*", "TDH*", "HYPER*", "ACTIV*", "ATENC*".

Table S2. Biennial Attention deficit hyperactivity disorder (ADHD)ases, person-years and incidence rate

**Year**	**2003**	**2005**	**2007**	**2009**	**2011**	**2013**	**2015**	**2017**	**2019**
**Cases**	322	697	921	1096	1199	1191	1091	766	108
**Person-years**	77011.97	100671.98	124289.90	147585.92	163726.17	171483.69	174236.52	159496.10	48975.61
**Incidence rate (per 1,000)**	4.18	6.92	7.41	7.43	7.32	6.95	6.26	4.80	2.21

Table S3. Goodness of fit of the age-period-cohort model[a]

	Deviance	Residual df	p	LR vs APC	Df vs APC	p	AIC
**APC**	78,302	35	<0.001	N/A	N/A	N/A	496.964
**AP**	100,741	48	<0.001	22.439	13	0.049	493.402
**AC**	468,173	42	<0.001	389.870	7	<0.001	872.834
**PC**	402,076	40	<0.001	323.773	5	<0.001	810.737
**Ad**	584,955	55	<0.001	506.653	20	<0.001	963.616
**Pd**	416,256	53	<0.001	337.953	18	<0.001	798.917
**Cd**	763,195	47	<0.001	684.892	12	<0.001	1157.856
**A**	585,225	56	<0.001	506.922	21	<0.001	961.886
**P**	496,451	54	<0.001	418.149	19	<0.001	877.112
**C**	823,981	48	<0.001	745.679	13	<0.001	1216.642
**t**	869,301	60	<0.001	790.999	25	<0.001	1237.963
**tA**	869,303	61	<0.001	791.000	26	<0.001	1235.964
**tP**	941,085	61	<0.001	862.783	26	<0.001	1307.746
**tC**	929,189	61	<0.001	850.887	26	<0.001	1295.851
**1**	944,405	62	<0.001	866.102	27	<0.001	1309.066

A: age; AC: age-cohort; Ad: age-drift; AIC: Akaike Information Criterion; AP: Age-period; APC: age-period-cohort; C: cohort; Cd: cohort-drift; d: drift (linear trend); df: degrees of freedom; LR: likelihood ratio; P: period; PC: period- cohort: trend; Pd: period-drift; N/A: not applicable; t: trend; tA: linear trend in ages; tC; linear trend in cohorts; tPp: linear trend in periods; 1: time-saturated model

[a]Table shows the fitting of APC model and all sub-models, and the results of the comparison between them using the Akaike Information Criterion (AIC). The most preferred model is that with the smallest AIC. Each row represents a model.

Table S4. Modeling of the effect of age, gender, year period and month on the incidence rate ratio of Attention deficit hyperactivity disorder (ADHD) (Poisson regression)[a]

**Predictors**	**Incidence Rate Ratios**	**CI**	**p**
age_ [06]	2.26	2.03 – 2.53	**<0.001**
age_ [07]	2.73	2.45 – 3.04	**<0.001**
age_ [08]	2.57	2.31 – 2.87	**<0.001**
age_ [09]	2.19	1.96 – 2.45	**<0.001**
age_ [10]	1.77	1.57 – 1.99	**<0.001**
age_ [11]	1.44	1.27 – 1.63	**<0.001**
age_ [12]	1.55	1.37 – 1.76	**<0.001**
age_ [13]	1.56	1.38 – 1.77	**<0.001**
age_ [14]	1.44	1.27 – 1.64	**<0.001**
age_ [15]	0.97	0.84 – 1.13	0.727
age_ [16]	0.71	0.60 – 0.84	**<0.001**
age_ [17]	0.53	0.43 – 0.65	**<0.001**
age_ [18]	0.29	0.22 – 0.38	**<0.001**
gender_ [male]	2.55	2.43 – 2.68	**<0.001**
year_ [2004]	1.82	1.45 – 2.30	**<0.001**
year_ [2005]	2.73	2.22 – 3.40	**<0.001**
year_ [2006]	2.19	1.77 – 2.73	**<0.001**
year_ [2007]	2.57	2.09 – 3.19	**<0.001**
year_ [2008]	2.94	2.40 – 3.64	**<0.001**
year_ [2009]	2.93	2.39 – 3.62	**<0.001**
year_ [2010]	3.03	2.48 – 3.75	**<0.001**
year_ [2011]	3.09	2.53 – 3.82	**<0.001**

year_ [2012]	3.60	2.96 – 4.44	**<0.001**
year_ [2013]	3.99	3.28 – 4.91	**<0.001**
year_ [2014]	3.39	2.78 – 4.18	**<0.001**
year_ [2015]	3.16	2.58 – 3.89	**<0.001**
year_ [2016]	2.88	2.36 – 3.56	**<0.001**
year_ [2017]	2.61	2.12 – 3.23	**<0.001**
year_ [2018]	2.71	2.20 – 3.36	**<0.001**
year_ [2019]	2.43	1.92 – 3.09	**<0.001**
month_ [02]	1.32	1.20 – 1.45	**<0.001**
month_ [03]	1.30	1.18 – 1.43	**<0.001**
month_ [04]	1.11	1.00 – 1.23	**0.040**
month_ [05]	1.17	1.06 – 1.29	**0.002**
month_ [06]	1.02	0.92 – 1.14	0.641
month_ [07]	0.48	0.42 – 0.55	**<0.001**
month_ [08]	0.57	0.51 – 0.65	**<0.001**
month_ [09]	0.70	0.62 – 0.79	**<0.001**
month_ [10]	0.92	0.83 – 1.02	0.130
month_ [11]	1.18	1.07 – 1.31	**0.001**
month_ [12]	1.05	0.94 – 1.16	0.385
Observations	5064		
R^2 Nagelkerke	0.653		

[a]Reference categories: year 2003, month 01 (January), age 5. Estimates >1 represent higher incidences.

CI: confidence interval

Table S5. Prevalence of Attention deficit hyperactivity disorder (ADHD) diagnosis according to calendar year, patients´ age at the time of ADHD diagnosis and gender

Calendar year	Age at the time of ADHD diagnosis (years)	Prevalence (per 100 persons)			p-Value
		Overall	Males	Females	
2007	5	0.15	0.15	0.16	1
	7	2.06	3.18	0.91	**<0.01**
	9	3.06	4.17	1.86	**<0.01**
	11	3.54	5.53	1.51	**<0.01**
	13	3.23	4.93	1.41	**<0.01**
	15	2.83	4.53	0.95	**<0.01**
	17	--	--	--	--
	19	--	--	--	--
2011	5	0.25	0.38	0.12	0.05
	7	2.00	2.93	0.99	**<0.01**
	9	4.88	6.75	2.96	**<0.01**
	11	5.98	8.19	3.74	**<0.01**
	13	5.66	7.87	3.28	**<0.01**
	15	5.68	8.83	2.47	**<0.01**
	17	4.77	7.27	2.11	**<0.01**
	19	3.14	4.96	1.13	**<0.01**
2015	5	0.15	0.24	0.06	0.11
	7	1.88	2.85	0.82	**<0.01**
	9	4.23	5.44	2.99	**<0.01**
	11	6.16	8.64	3.47	**<0.01**
	13	7.31	10.28	4.24	**<0.01**
	15	8.19	11.46	4.87	**<0.01**
	17	7.57	10.66	4.26	**<0.01**
	19	6.48	9.71	3.19	**<0.01**
2019	5	--	--	--	--
	7	--	--	--	--
	9	2.94	4.34	1.45	**<0.01**
	11	4.31	6.20	2.24	**<0.01**
	13	5.63	7.34	3.91	**<0.01**
	15	7.44	10.31	4.30	**<0.01**
	17	8.52	12.01	4.90	**<0.01**
	19	8.74	12.05	5.37	**<0.01**

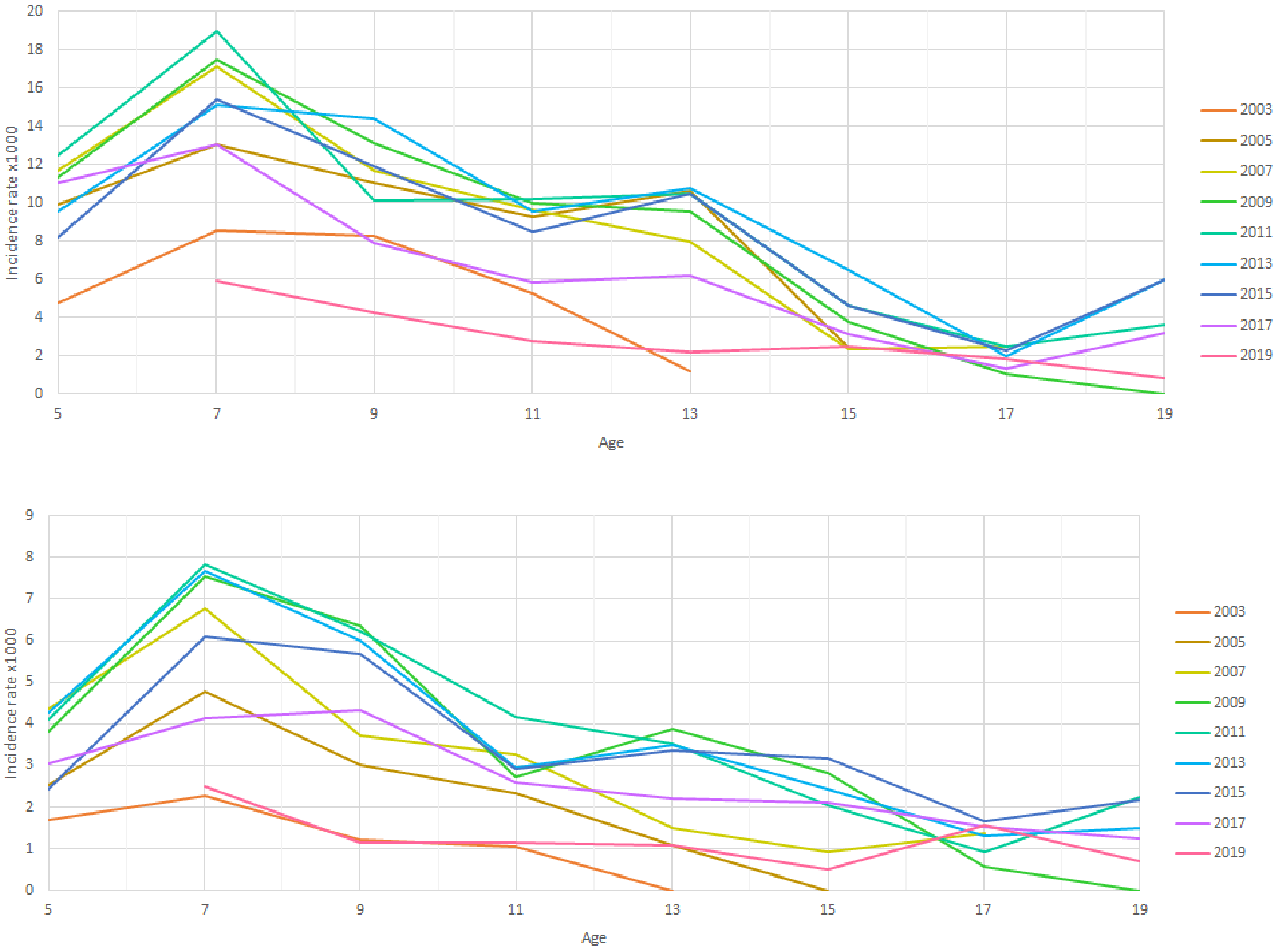

Figure S1. Incidence of Attention deficit hyperactivity disorder (ADHD) diagnosis according to age at the time of ADHD diagnosis and calendar year. <u>Top</u>: **MALES**. <u>Bottom</u>: **FEMALES**

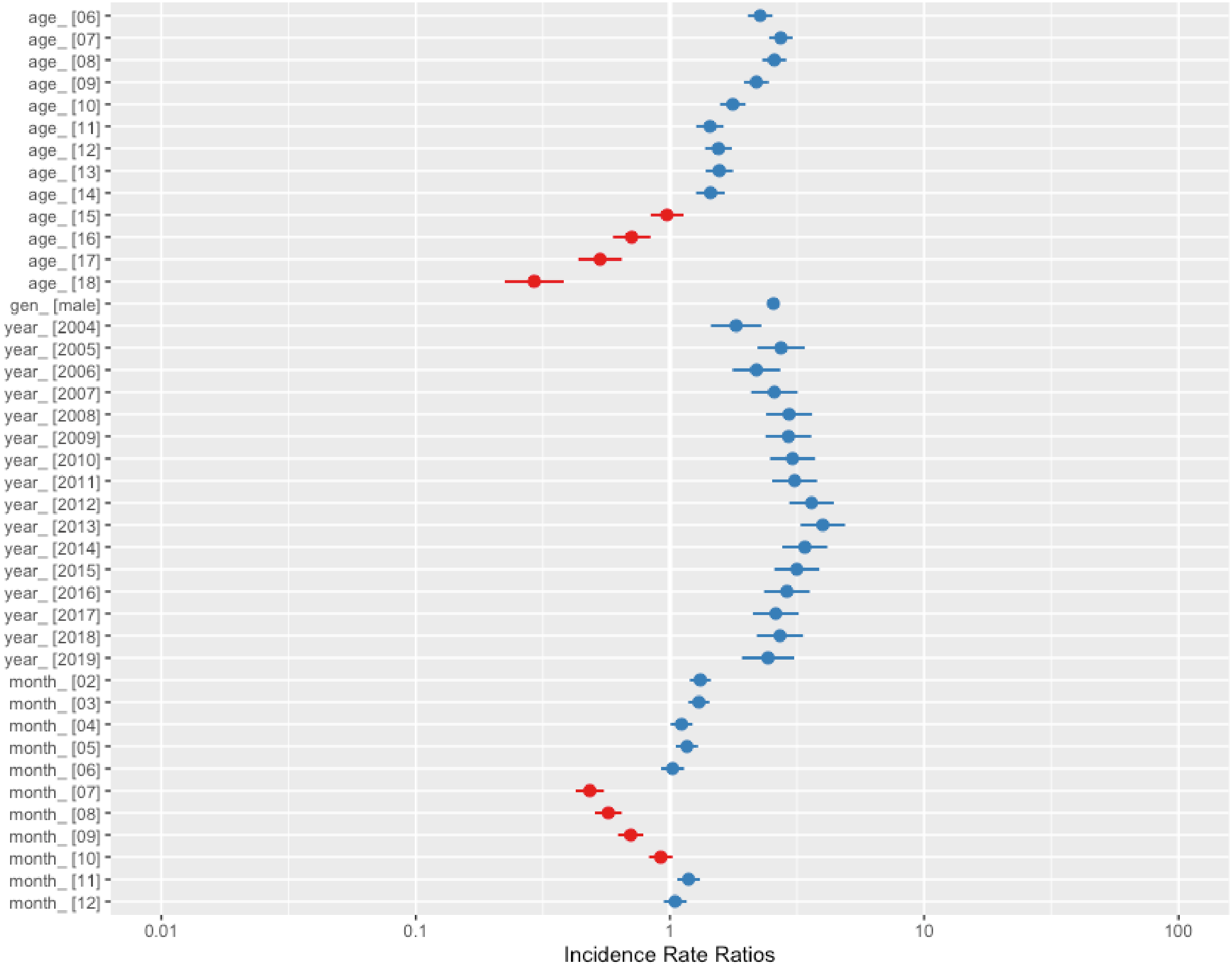

Figure S2. Modeling of the effect of age, gender, year period and month on the incidence rate ratio of Attention deficit hyperactivity disorder (ADHD) (Poisson regression). Reference categories: year 2003, month 01 (January), age 5. Estimates >1 represent higher incidences.

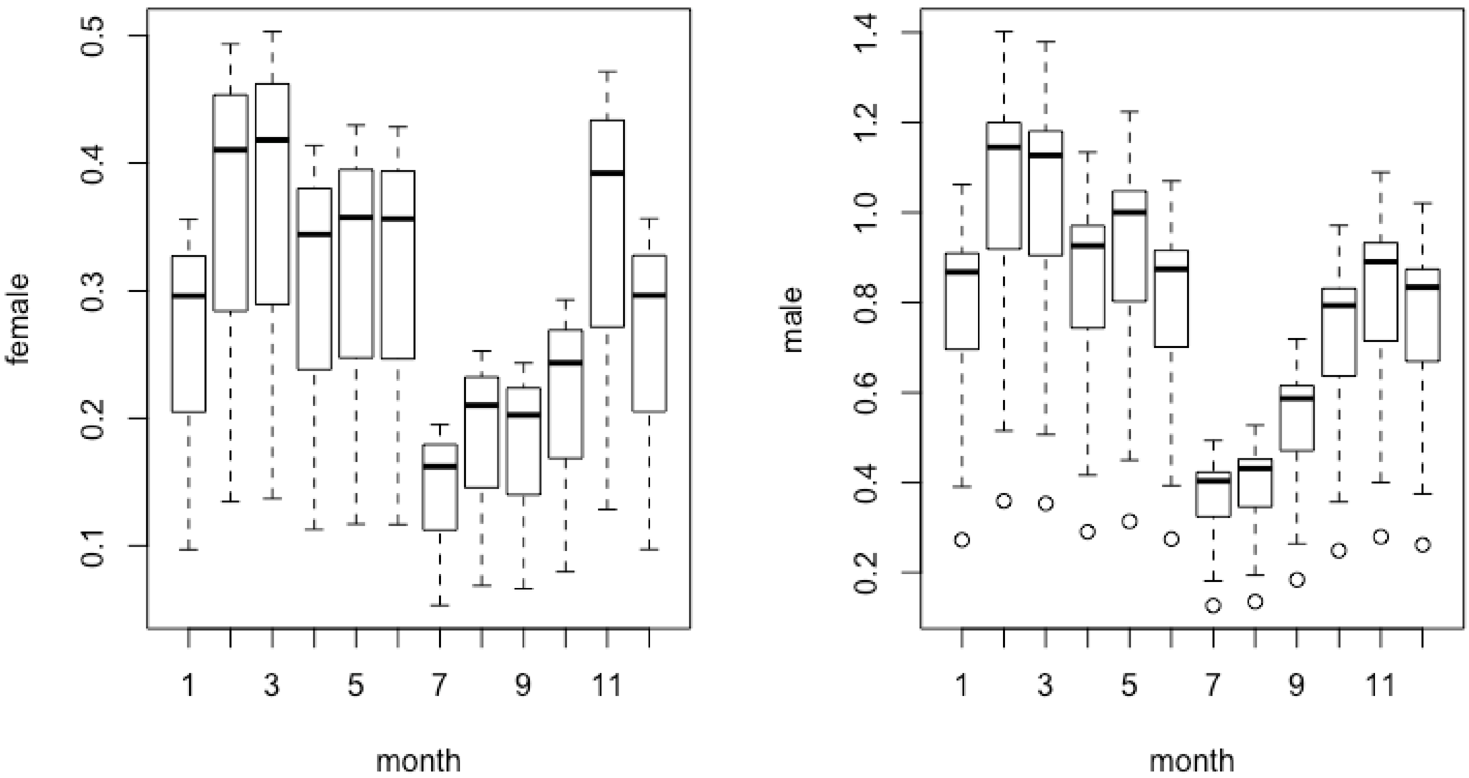

Figure S3. Seasonality of Attention deficit hyperactivity disorder (ADHD) incidence. Left: **FEMALES**. Right: **MALES**

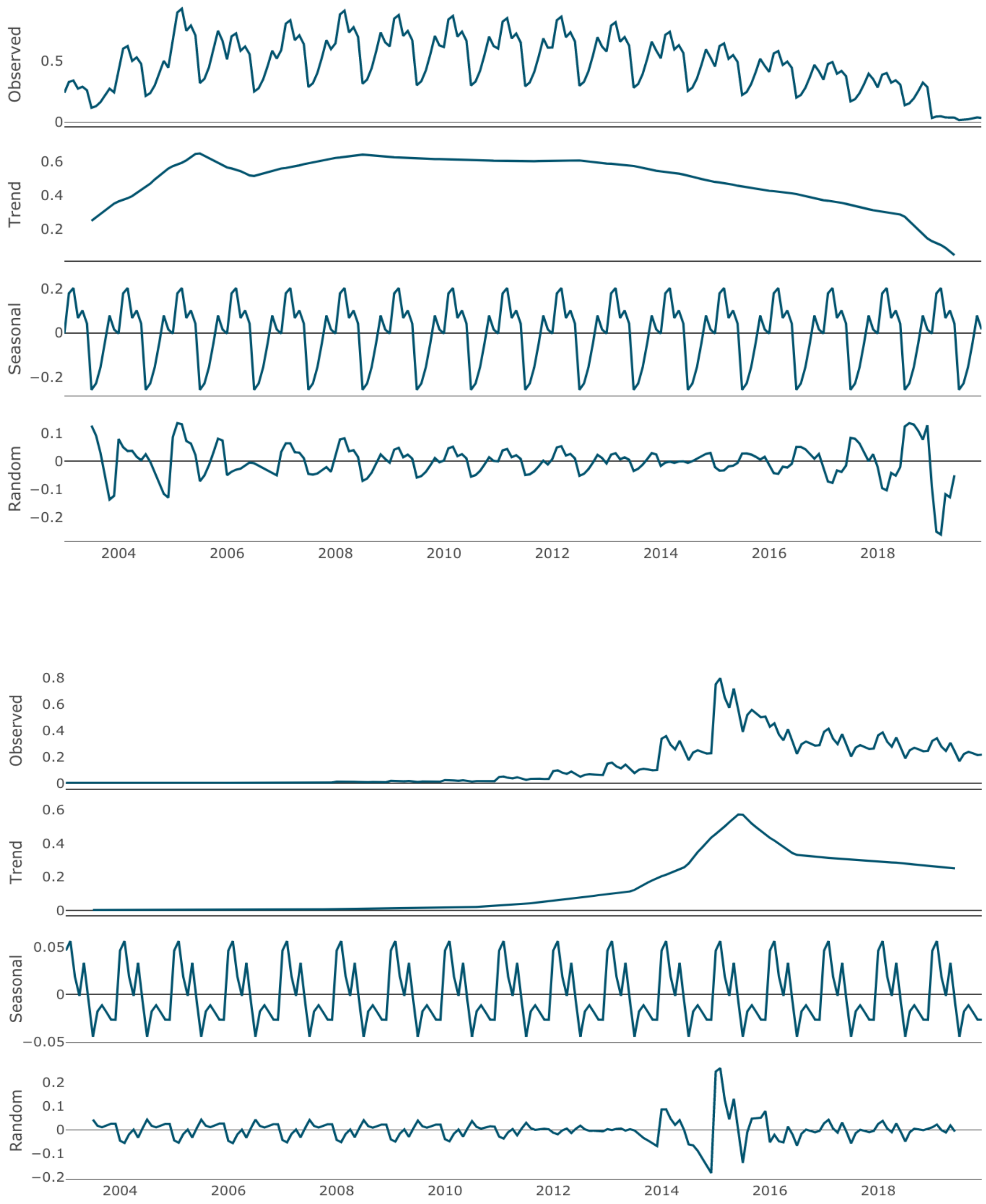

Figure S4. Decomposition of monthly time series of the standardized Attention deficit hyperactivity disorder (ADHD) incidence rate by calendar year (both males and females) (2019 data up to 20 November). Top: ADHD diagnoses from **PRIMARY CARE.** Bottom: ADHD diagnoses from **SPECIALIZED CARE**

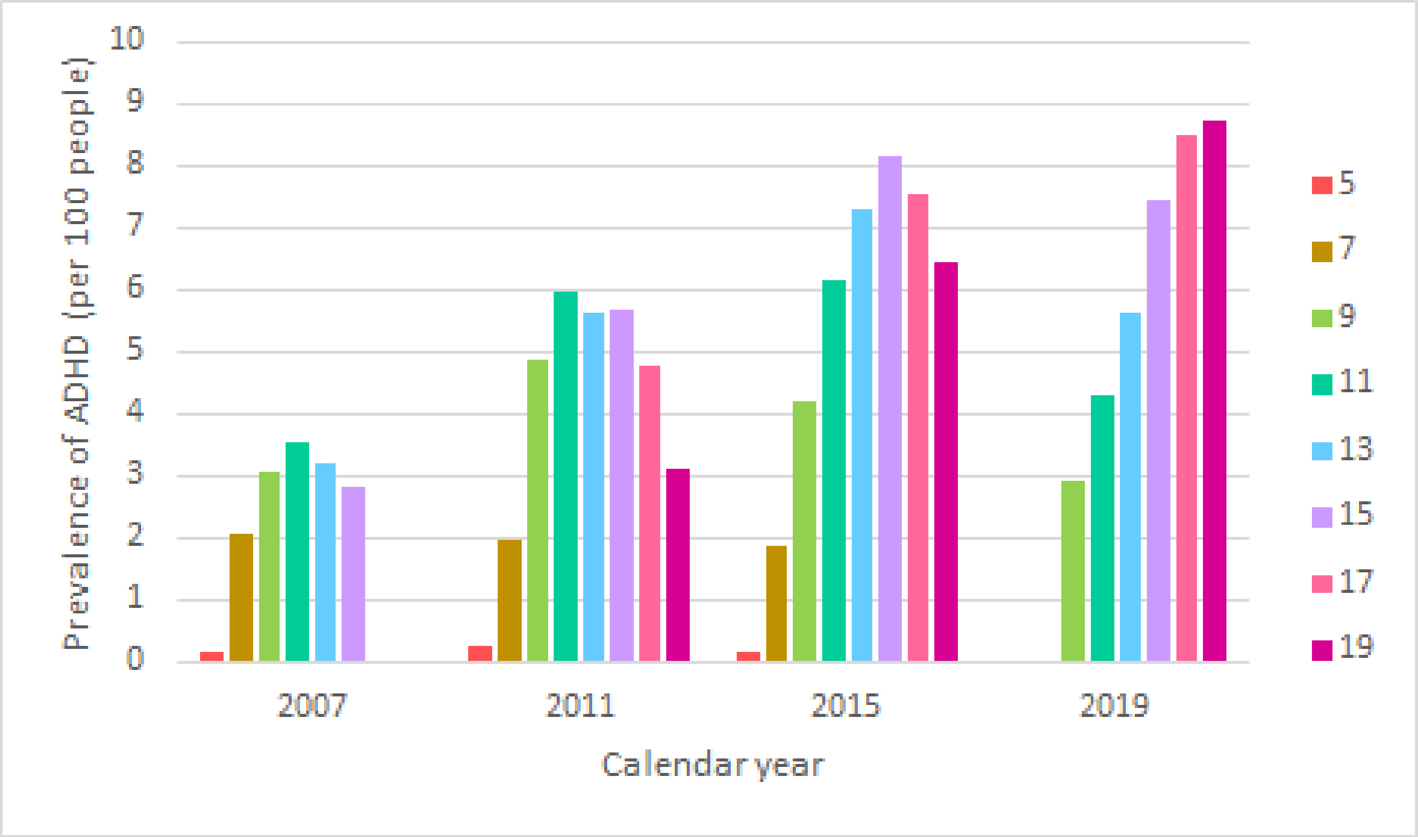

Figure S5. Prevalence of Attention deficit hyperactivity disorder (ADHD) diagnoses according to calendar year and patients´ age at the time of ADHD diagnosis (both males and females)
